# Performance of AFEX™ pretreated rice straw as source of fermentable sugars: the influence of particle size

**DOI:** 10.1186/1754-6834-6-40

**Published:** 2013-03-21

**Authors:** Shuhaida Harun, Venkatesh Balan, Mohd Sobri Takriff, Osman Hassan, Jamaliah Jahim, Bruce E Dale

**Affiliations:** 1 Department of Chemical and Process Engineering, Faculty of Engineering and Built Environment, Universiti Kebangsaan Malaysia, Bangi, Selangor, 43600 UKM, Malaysia; 2 Department of Chemical Engineering and Materials Science, DOE Great Lakes Bioenergy Research Center, Michigan State University, Lansing, MI, 48823, USA; 3 School of Chemical Sciences and Food Technology, Faculty of Science and Technology, Universiti Kebangsaan Malaysia, Bangi, Selangor, 43600 UKM, Malaysia

**Keywords:** Lignocellulosic biomass, Rice straw, Particle size, AFEX pretreatment, Compositional analysis, Large particle, Enzymatic hydrolysis, Conversion, Yield, Ethanol production

## Abstract

**Background:**

It is widely believed that reducing the lignocellulosic biomass particle size would improve the biomass digestibility by increasing the total surface area and eliminating mass and heat transfer limitation during hydrolysis reactions. However, past studies demonstrate that particle size influences biomass digestibility to a limited extent. Thus, this paper studies the effect of particle size (milled: 2 mm, 5 mm, cut: 2 cm and 5 cm) on rice straw conversion. Two different Ammonia Fiber Expansion (AFEX) pretreament conditions, AFEX C1 (low severity) and AFEX C2 (high severity) are used to pretreat the rice straw (named as AC1RS and AC2RS substrates respectively) at different particle size.

**Results:**

Hydrolysis of AC1RS substrates showed declining sugar conversion trends as the size of milled and cut substrates increased. Hydrolysis of AC2RS substrates demonstrated opposite conversion trends between milled and cut substrates. Increasing the glucan loading to 6% during hydrolysis reduced the sugar conversions significantly in most of AC1RS and AC2RS except for AC1RS-2 mm and AC2RS-5 cm. Both AC1RS-2 mm and AC2RS-5 cm indicated gradual decreasing trends in sugar conversion at high glucan loading. Analysis of SEM imaging for URS and AFEX pretreated rice straw also indicated qualitative agreement with the experimental data of hydrolysis. The largest particle size, AC2RS-5 cm produced the highest sugar yield of 486.12 g/kg of rice straw during hydrolysis at 6% glucan loading equivalent to 76.0% of total theoretical maximum sugar yield, with an average conversion of 85.9% from total glucan and xylan. In contrast, AC1RS-5 cm gave the lowest sugar yield with only 107.6 g/kg of rice straw, about 16.8% of total theoretical maximum sugar yield, and equivalent to one-quarter of AC2RS-5 cm sugar yield.

**Conclusions:**

The larger cut rice straw particles (5 cm) significantly demonstrated higher sugar conversion when compared to small particles during enzymatic hydrolysis when treated using high severity AFEX conditions. Analysis of SEM imaging positively supported the interpretation of the experimental hydrolysis trend and kinetic data.

## Background

Lignocellulosic biomass (LCB) consisting of cellulose, hemicellulose and lignin, such as agricultural residues, woody materials, energy crops and perennial crops, is a promising feedstock mainly because of its low cost, abundant availability and low environmental impacts. Commercialization of biofuels from LCB will create local job markets, improve local economic development and reduce greenhouse gas emissions when compared to fossil fuels [1-3].

Among the crop residues of LCB, rice straw is one of the most plentiful crop residues in the world, and is produced at the rate of approximately 731 million tonnes per year with Asia as the largest producer at about 667.6 million tonnes. This amount of rice straw can potentially produce 205 billion litres of bioethanol annually and would become the world’s largest single biomass feedstock source of bioethanol [4,5]. About 60% of the mass of the rice crop production is rice straw, and it is composed of leaf and sheath (53%), stem (44%) and panicles (3%) when cut at ground level [6]. Customarily, most farmers in the world openly burn rice straw since this practice offers a cost effective method for disposing of the straw and clearing the rice field for planting the next crop [7]. However, this practice creates serious environmental, safety and health issues, and there is a strong desire to find alternative ways to remove the rice straw after each harvesting season. Recent research findings on producing biofuels and high value reactive intermediates such as fermentable sugars from LCB have provided new options for farmers wishing to be more environmentally friendly while adding an extra source of income [4,5,8].

Conversion of LCB to biofuels such as ethanol is more challenging than starchy material, such as corn, owing to the complex and recalcitrant structure of the plant cell wall [2]. Unlike corn, where starch carbohydrates are easily depolymerized into fermentable sugars, carbohydrate fractions in LCB (cellulose and hemicellulose) are not readily available for enzymatic hydrolysis. The accessibility of enzymes to cellulose and hemicellulose in untreated LCB is a major hurdle in biochemical conversion technology [2,9-11]. Hence, pretreatment is an essential processing step required to improve accessibility of the enzymes to the cellulose and hemicellulose. An effective pretreatment should open up the LCB cell wall matrix, hydrolyse the hemicelluloses, reduce cellulose crystallinity and ultimately make the cellulose and hemicellulose more accessible to the enzymes in the subsequent hydrolysis process that converts the carbohydrate polymers into fermentable sugars [2,3,10].

Ammonia Fiber Expansion (AFEX) is one of the leading pretreatment technologies available that offers an effective and economically attractive means of increasing the yields of fermentable sugars from LCB [12]. AFEX has been highly successful in opening up the cell wall in agricultural residues [13], de-crystallization of cellulose, partial de-polymerization of hemicellulose, de-acetylation of acetyl groups [14], and cleavage of the lignin carbohydrate complex (LCC) with greatly reduced degradation products when compared to acidic pretreatments [1,15]. Studies have shown that AFEX pretreatment helps improve enzymatic digestibility several fold over untreated LCB [16,17]. In the AFEX process, biomass is pretreated with liquid ammonia at moderate temperatures and high pressure for a specific residence time. The pressure is then rapidly released, literally expanding the fibrous biomass. The ammonia evaporates readily and over 97% of it can be recovered and reused. The resulting AFEX pretreated biomass is recovered completely since there is no wash stream and can be readily hydrolysed at near theoretical yields of fermentable sugars [1,17-19].

In addition to chemical pretreatment, physical pretreatment of LCB such as grinding, milling or chipping is recommended for particle size reduction [3]. The goal of this size reduction is to reduce the crystallinity of the cellulose fibers in the biomass [10]. Size reduction of LCB is also reported to be necessary to eliminate mass and heat transfer limitations during pretreatment and enzymatic hydrolysis [20]. Most of the previous studies on pretreated rice straw and other LCBs focused on small particle size, which is normally less than 5 mm [1,19,21]. Very extensive size reduction is undesirable since the grinding and milling of biomass is an energy-intensive and very expensive process [21,22] and also causes significant carbohydrate losses which ultimately results in less reducing sugars and a reduction in ethanol yield [21]. Previous work on the influence of larger particle size in the biomass conversion process is limited. As biomass to biofuel technologies near the commercializing stage, processing with larger particle size could significantly improve the energy cost due to excessive grinding process.

The complexity of the enzymatic hydrolysis of LCB stems from the fact that it is a heterogeneous insoluble substrate and thus enzymatic hydrolysis is always limited by access to available surfaces. In a heterogeneous system it is possible to study enzymatic hydrolysis kinetics using time course data [23,24]. Also, it is possible to consider that these enzymatic reactions are diffusion limited and therefore the hydrolysis time curves depend strongly on the heterogeneous rate-limiting structures of the substrate–enzyme system. Eq. (1) shows the diffusion-limited kinetic model proposed by Chrastil [23,24]. In this model, there are two factors determining the behaviour of the system: initial enzyme concentration and the equilibrium product concentration. Eq. (1) is given as below:

(1) $$P=P_{e}{\left(1-e^{-kE_{o}t}\right)}^{n}$$

where *P* and *P*_*e*_ are the product concentrations at every considered time *t* and at equilibrium, respectively, *k* is a rate constant proportional to the diffusion coefficient as defined by Fick’s law, *E*_*o*_ is the initial enzyme concentration and *n* is a structural diffusion resistance constant depending on the steric features of the system. The parameter *n* defines the reaction order characteristics. When diffusion resistance is small, *n* tends to 1 (for low-resistance films *n* = 0.9–1.0) and the reaction is of apparent first order. If the system is strongly limited by diffusion resistance, *n* is small (high-resistance structures *n* = 0.5–0.6). In addition, when *n* > 1, a consecutive reaction order may be expected [23].

In this study we explored the effect of two different AFEX pretreatment severities on different particle sizes of rice straw (as small as 2 mm to as large as 5 cm). We also conducted the compositional analysis of the untreated and pretreated rice straw. Subsequently, we performed enzymatic hydrolysis at different glucan loadings to compare digestibility, sugar conversions and yields of the pretreated rice straw at different particle sizes. We fitted the enzymatic hydrolysis data for each particle size into the Chrastil kinetic model to determine the kinetic parameters and carried out SEM imaging in order to explain the effect of AFEX pretreatment conditions on the hydrolysis kinetics at different particle sizes.

## Results and discussion

### Compositional analysis of untreated and AFEX pretreated rice straw

The major structural components of biomass feedstocks are cellulose (glucan), klason lignin and hemicellulose, primarily made up of xylan. Other sugars and lignins that make up the structural component are galactan, arabinan, mannan, acetyl groups and acid soluble lignin. Non-structural components that are generally measured are extractives and proteins [25,26]. Table 1 presents the compositions of UTRS and AFEX pretreated rice straw. In general, the compositions of the structural components of the UTRS were made up of structural carbohydrates (57.8%), Klason lignin (19.8%), and acetyl groups (1.6%). The carbohydrates were composed of glucan, xylan and arabinan (34.4%, 19.7% and 3.7%, respectively). The non-structural components of the UTRS accounted for about 21.2% of the rice straw; they were comprised primarily of ash, extractives and nitrogen.

**Table 1:** The compositions of UTRS, AC1RS and AC2RS

**Components**	**Composition of rice straw (expressed as the percentage of oven dried, %)**		
	**UTRS**	**AC1RS**	**AC2RS**
**Structural components:**			
1. Structural carbohydrates	57.8 ± 0.6	57.2 ± 0.3	57.8 ± 0.4
Glucan	34.4 ± 0.6	33.8 ± 0.2	34.6 ± 0.4
Xylan	19.7 ± 0.2	19.8 ± 0.2	19.5 ± 0.1
Arabinan	3.7 ± 0.1	3.6 ± 0.0	3.7 ± 0.1
2. Lignin	19.8 ± 0.8	15.4 ± 0.8	15.8 ± 1.0
3. Acetyl group	1.6 ± 0.1	1.4 ± 0.1	1.7 ± 0.0
**Non-structural components:**			
1. Ash	14.1 ± 0.2	13.5 ± 0.1	13.4 ± 0.2
2. Nitrogen (native)	0.5 ± 0.2	0.5 ± 0.2	0.5 ± 0.2
3. Nitrogen (AFEX)	NA	2.5 ± 0.4	3.7 ± 0.4
4. Extractives	6.7 ± 1.8	11.8 ± 1.4	12.8 ± 1.0

All values are means of duplicate ± standard deviation; NA – Not applicable.

The structural carbohydrates of AC1RS and AC2RS were 57.2% and 57.8%, respectively, and were composed of approximately 33.8-34.6% glucan, 19.5-19.8% xylan and 3.6-3.7% arabinan. A statistical paired t-test on the mean composition of UTRS and AFEX pretreated rice straw (AC1RS and AC2RS) indicated that the differences in compositions of carbohydrate components (glucan, xylan and arabinan), acetyl groups, and ash were statistically insignificant (t-stat < t_critical_ and p > 0.05). This was due to the “dry to dry” AFEX process, which prevents the loss of holocellulosic components during pretreatment of rice straw [17,27,28].

The compositions of lignin, nitrogen and extractives between UTRS and AFEX pretreated rice straw showed significant differences (t-stat > t_critical_ and p < 0.05). The decrease in lignin of AC1RS and AC2RS was potentially due to the lignin degradation during the AFEX pretreatment, which was solubilized and re-deposited on the biomass surface [1]. During the two-stage acid hydrolysis step of compositional analysis, this newly re-deposited lignin would be released in the form of acid soluble lignin [29] which is indicated by an increase in the total extractives. The increase in nitrogen of AC1RS and AC2RS was mainly due to the addition of ammonia to the biomass during the AFEX pretreatment itself. Previous work on AFEX pretreatment of several biomass types, including rice straw, also indicated a similar trend of compositional changes in the pretreated materials [1,19].

Hemicelluloses of rice straw are characterized experimentally and are comprised primarily of α–L-(1–3)-arabino-(4-O-methyl-α-(1–2)-D-glucurono)-β-(1–4)-D-xylan and arabino-glucuronoxylan (AGX) [30]. The xylan backbone β-(1–4)-D-xylopyranosyl units are substituted by monomeric 4-O-methyl-α-D-glucopyranosyl uronic acid residue (4-O-MeGlcA) and an α–L-arabinofuranosyl unit at the C_2_ and/or C_3_ main chain. A significant portion of the xylose in cereal straw cell walls is acetylated, mainly on C_2_ and C_3_, and the acetyl groups account for 1-2% [31,32]. Lignin exists in plant tissue as a dependent polymer and is always associated with cellulose, hemicelluloses and other polymers as lignin-carbohydrate complexes (LCCs) through covalent bonds. In herbaceous plants like rice straw, LCCs contain ferulic bridges which are attached to lignin and carbohydrates (AGX) via ether and ester bonds, respectively. Alkali cleaves the ester bond components of such bridges, liberating the ferulic acid (FA) residue and lignin from carbohydrates and yielding a small amount of FA (1-4%) [30,33,34]. Experimental analysis on isolated LCCs from rice straw reveals that it contains 64% carbohydrates, 3% uronic acid, 33% lignin, 4% acetyl groups, 4% trans-p-coumaric acid and 1% trans-ferulic acid [35].

The AFEX C2 condition yielded more nitrogen in pretreated rice straw (3.7%) when compared to the AFEX C1 condition (2.5%) (Table 1). This finding is interesting, as the ratio of ammonia to solid in the AFEX C2 condition (1:1) was half that of the AFEX C1 condition (2:1). This may indicate that with the AFEX C2 condition, where higher reaction temperature (140°C) was applied, more ammonia was able to penetrate the cellulose, resulting in the formation of ammonia-cellulose complexes. This led to the incorporation of ammonia into the cellulose crystal lattice, causing lattice transformation and crystal plane widening [36], a known swelling effect [27,37].

During AFEX pretreatment, the incorporated ammonia has the tendency to cleave the ester linkages of AGX via ammonolysis [38] and hydrolysis reactions in the rice straw. The increase in the total extractives of AFEX pretreated rice straw, AC1RS and AC2RS, after water and ethanol extractions indicates that AFEX pretreatment was able to chemically cleave the structure of lignin and AGX in LCCs [33] and these hemicelluloses and lignin residues were easily extracted and solubilized in the subsequent solvent extractions. The total extractives extracted out of the rice straw, including the water soluble products, acid soluble lignin, soluble proteins, soluble salts and minerals, and others, significantly increased with increasing pretreatment severity, from 14.0% in UTRS to 25.3% in AC1RS and 30.2% in AC2RS. This implies the presence of additional solubilized substituents from the pretreated rice straw. These results were consistent with previous reports [15,38].

**Figure 1:**
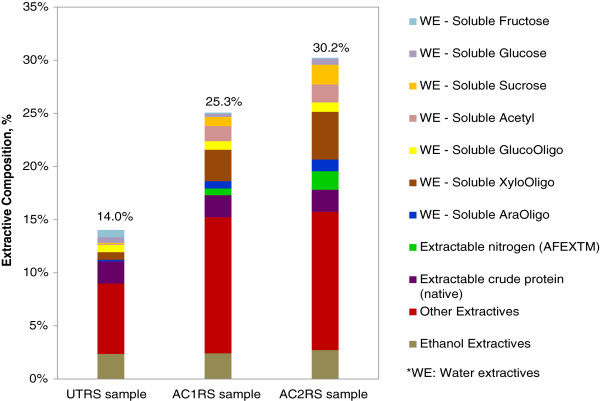
Composition of extractives of UTRS and AFEX pretreated rice straw-AC1RS, AC2RS.

Figure 1 characterizes the composition of the total extractives in water and ethanol extractions. Cleavage of LCC was supported by an increase in soluble oligomeric sugars found in water extractions of AFEX pretreated samples. In comparison to UTRS water extraction, AC1RS and AC2RS water extraction yielded 4.1 and 6.2 fold-increases of soluble xylose oligomers, 4.1 and 7.3 fold-increases of soluble arabinose oligomers as well as 16 and 19 fold-increases of soluble acetyl groups, respectively (Figure 1). This increase in soluble acetyl groups is likely due to the dissolution of the O-acetyl linkage on the xylan-pyranose backbone side chain via ester bond breakage in this alkaline treatment.

In general, xylan in cell walls of graminaceous plants, like rice straw, is composed of 1-2% O-acetyl groups [25,39]. Hemicellulose components, xylose, arabinose and acetyl dissolved and solubilized more during water extraction of AC2RS compared to AC1RS, showing more occurrence of structural disruptions under more severe AFEX C2 conditions. Previous studies show that corn stover with severe AFEX pretreatment results in a 50% increase in the total water extractives when compared to corn stover undergoing more moderate AFEX pretreatment. Release of arabinoxylan oligomers accounts for this significant increase. The release of lignin degradation products such as vanillin, syringic acid and homovanilic acid shows positive correlation (>25% increase) to increasing AFEX pretreatment severity [15]. Although lignin degradation products were not quantified in this study, they were partially accounted for in the fraction of other extractable materials (classified as other extractives in Figure 1).

Other un-quantified extractives may include gums, resins, pitch, waxes, sterols, flavinoids, tannins, terpenes, quinones, non-structural sugars, chlorophyll and other minor building blocks [40]. It was observed that the fraction of other extractives (non-quantified components) was higher in both AC1RS and AC2RS extractions when compared to UTRS extraction. The other extractives in both AC1RS and AC2RS extractions were 12.8% and 13.0%, respectively, while in the UTRS extraction they were only 6.6% (Figure 1).

### Enzymatic hydrolysis and kinetic modelling of AFEX pretreated rice straw

#### Low solid loading hydrolysis (1% glucan loading) - monomeric sugar release

**Figure 2:**
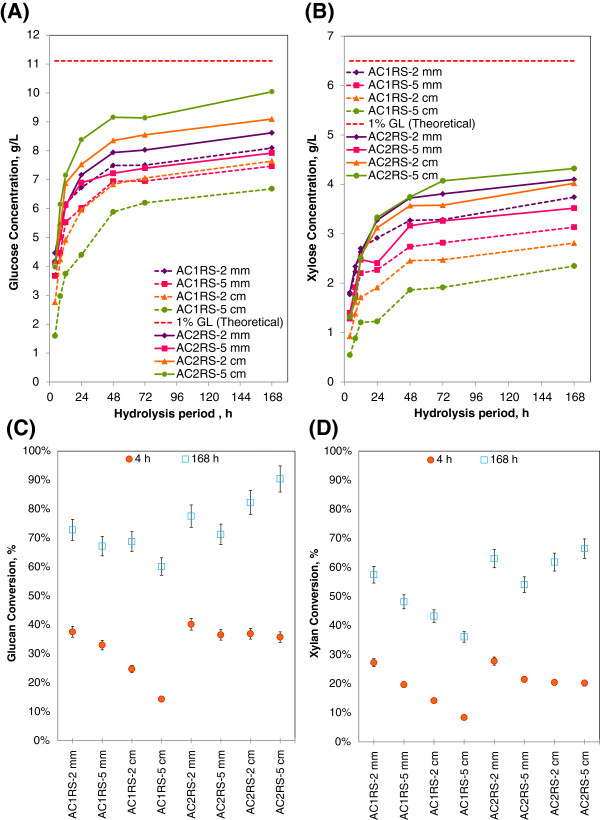
**Monomeric sugar concentrations and conversion profiles at 1% glucan loading for AFEX C1 (AC1RS) and AFEX C2 (AC2RS) at different hydrolysis periods and biomass sizes – 15 mL, Novozyme and Spezyme CP, 50 °C and 150 rpm.** (**A**) & (**B**) – Glucose & xylose concentrations, (**C**) & (**D**) – Glucan & xylan conversions.

Figure 2(A)/2(B) elucidates the time course of glucose monomer (A) and xylose monomer (B) concentrations obtained from 1% glucan loading enzymatic hydrolysis of AC1RS and AC2RS substrates at different particle sizes. The maximum theoretical sugar concentrations are indicated by the red dashed line at the top of each figure. Glucose and xylose were rapidly released at the beginning of the process, and then the sugar generation rate slowed down as hydrolysis proceeded, as reported by other researchers [41]. Approximately 50-80% of the total glucose and xylose released was liberated from glucan and xylan of pretreated rice straw within the first 12 h of hydrolysis at 1% glucan loading. The simplified model of enzymatic hydrolysis divides the hydrolysis into two stages: the initial stage, where the rate is almost linear and the final stage, where the rate continuously decreases [42].

At 1% glucan loading hydrolysis, AC1RS substrates hydrolysed at a low initial hydrolysis rate during the first 8 h (linear slope), and the rates ranged from 0.37 g/L.h (AC1RS-5 cm) to 0.62 g/L.h (AC1RS-2 mm). Hence, this slow hydrolysis of AC1RS substrates produced low glucose and xylose concentrations at the end of 168 h hydrolysis (Figure 2(A)/2(B)). Among AC1RS substrates, only milled rice straw of AC1RS-2 mm produced the highest final glucose concentration with 8.1 g/L. Milled rice straw of AC1RS-5 mm and AC1RS-2 cm gave approximately similar glucose concentrations: 7.5 g/L and 7.6 g/L, respectively. Finally the largest particles size of AC1RS, AC1RS-5 cm, ended up with the lowest glucose concentration of 6.7 g/L. A similar decreasing trend was also observed for the xylose concentration for all AC1RS substrates.

A different trend of sugar production was observed in the hydrolysis of most AC2RS substrates at the same glucan loading (Figure 2(A)/2(B)). AC2RS substrates quickly hydrolysed during the first 8 h with the initial hydrolysis rate ranging from 0.62 g/L.h (AC2RS-5 mm) to 0.77 g/L.h (AC2RS-5 cm). As a result, this fast hydrolysis of AC2RS substrates produced higher glucose and xylose concentrations at the end of 168 h hydrolysis when compared to AC1RS substrates (except for AC2RS-5 mm where the concentration slightly dropped after 24 h). AC2RS-5 cm gave the highest glucose production when treated using the AFEX C2 condition with glucose concentration of 10.0 g/L. AC2RS-2 cm had a slightly lower concentration of 9.2 g/L. However, AC2RS milled rice straw (AC2RS-2 mm and AC2RS-5 mm) had lower glucose concentrations of 8.6 g/L and 7.9 g/L, respectively. AC2RS-5 cm also produced the highest xylose concentration with 4.3 g/L, almost a 2 fold increase when compared to AC1RS-5 cm, while AC2RS- 2 cm gave 4.0 g/L. Both 2 mm and 5 mm milled rice straw did not really show any significant difference in xylose concentrations when pretreated using either the AFEX C1 or C2 condition.

Figure 2(C)/2(D) shows the glucan (C) and xylan (D) conversion at 1% glucan loading hydrolysis at the 4 h and 168 h hydrolysis period. In both figures, AC1RS substrates showed a declining sugar conversion trend as the size of milled and cut substrates increased (i.e., milled: AC1RS-2 mm > AC1RS-5 mm, cut: AC1RS-2 cm > AC1RS-5 cm). After 168 h hydrolysis of AC1RS substrates, AC1RS-2 mm produced the highest glucan and xylan conversions with 72.8% and 57.5%, respectively, while AC1RS-5 cm gave the lowest glucan and xylan conversions at only 60.2% and 36.1%, respectively. Hydrolysis of AC2RS substrates demonstrated an opposite conversion trend between milled and cut substrates. Milled AC2RS substrates showed a decreasing sugar conversion trend as the size increased (i.e., AC2RS-2 mm > AC2RS-5 mm) which was similar to milled AC1RS substrates. Interestingly, for cut AC2RS substrates an increasing sugar conversion trend was noticed as the size increased (i.e., AC2RS-2 cm < AC2RS-5 cm). After 168 h hydrolysis, the largest particle size rice straw (AC2RS-5 cm) produced the highest glucan and xylan conversions, at 90.4% and 66.5%, respectively, when compared to the other particle size of AC2RS substrates pretreated under identical conditions.

**Figure 3:**
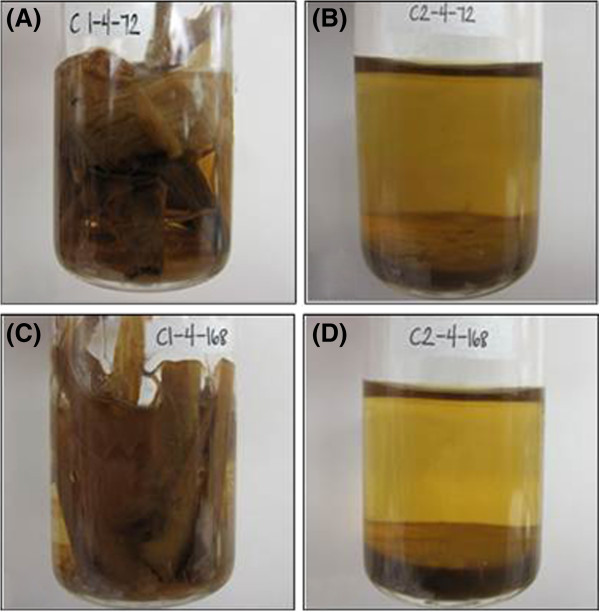
**Enzymatic hydrolysis of 1% GL of AC1RS-5 cm and AC2RS - 5 cm.** (**A**) AC1RS – 5 cm after 72 h; (**B**) AC2RS – 5 cm after 72 h; (**C**) AC1RS – 5 cm after 168 h; (**D**) AC2RS – 5 cm after 168 h.

The largest particle rice straw (5 cm) demonstrated qualitatively different digestion patterns during enzymatic hydrolysis when pretreated using different AFEX conditions. The substrate of AC1RS-5 cm hydrolysed slowly and the particles remained intact with minor physical disintegration even after 168 h of hydrolysis (Figure 3), evidenced by low sugar concentrations in the hydrolysate. The substrate of AC2RS-5 cm completely disintegrated after the same period of hydrolysis and only left fine particles in the hydrolysate (Figure 3), resulting in the highest sugar concentrations and therefore higher glucan and xylan conversions. In contrast, both sizes of milled rice straw (2 mm and 5 mm), when pretreated using AFEX C1 and AFEX C2 conditions, did not manifest any significant differences physically during hydrolysis nor in the sugar production.

### Low solid loading hydrolysis (1% glucan loading) - oligomeric sugar release

**Figure 4:**
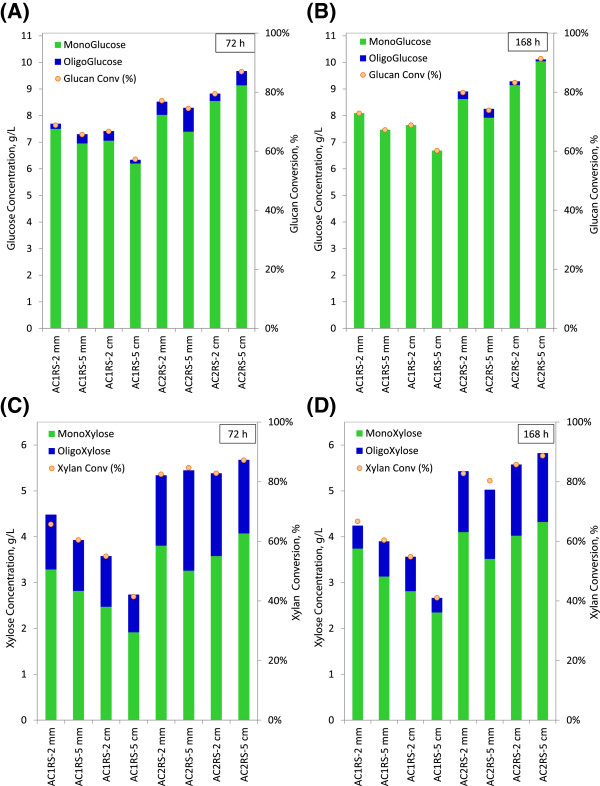
**Comparison of monomeric and oligomeric sugar conversion at 1% glucan loading for AFEX C1 (AC1RS) and AFEX C2 (AC2RS) at different biomass sizes.** (**A**) & (**B**) – Glucose concentration/glucan conversion at 72 h and 168 h, (**C**) & (**D**) – Xylose concentration/xylan conversion at 72 h and 168 h.

Figure 4(A)/4(B) and Figure 4(C)/4(D) compare monomeric and oligomeric glucose/xylose levels after 72 h and 168 h hydrolysis for both AC1RS and AC2RS substrates. From these comparison plots, a few observations can be drawn. First, more oligomers of glucose and xylose (higher concentrations and conversions) were observed in AC2RS substrates when compared to AC1RS substrates (both after 72 h and 168 h of hydrolysis), indicating the effectiveness of the AFEX pretreatment conditions (AFEX C2 over AFEX C1). Second, increasing concentrations of monomeric sugars and decreasing amounts of oligomeric sugars are evidenced as the hydrolysis proceeds (from 72 h to 168 h). Third, oligomeric xylose concentrations for AC1RS and AC2RS substrates were much higher when compared to oligomeric glucose concentrations, averaging at least 4 fold greater than oligomeric glucose concentrations (Figure 4(A)/4(C) for 72 h and Figure 4(B)/4(D) for 168 h of hydrolysis). Most xylose was released in oligomeric form, consistent with data reported for hemicellulose hydrolysis by others [43].

In this study, the combination of Spezyme CP and Novozyme 188 could not hydrolyse the oligomeric xylose to monomeric xylose which led to the high concentration of oligomeric xylose and low concentration of monomeric xylose. This was potentially caused by the insufficient β-xylosidase activity in these commercial enzymes. Previous work by Qing and Wyman showed that although Spezyme CP and Novozyme 188 preparations contained hemicellulolytic activities such as xylanase and β-xylosidase activities, the hydrolysis of the oligomeric xylose using these enzymes preparations still left significant amounts of higher degree of polymerization (DP) oligomeric xylose in the hydrolysis broth due to comparatively low β-xylosidase activity. They showed that supplementation with Multifect xylanase could not hydrolyse long chain oligomeric xylose, but addition of β-xylosidase nearly eliminated all oligomeric xylose in the hydrolysis broth [43]. Overall, after 72 h (168 h) hydrolysis, the highest glucose/xylose concentrations and glucan/xylan conversions (including the oligomeric sugar) were found to be 9.68/5.68 g/L (10.11/5.82 g/L) and 87.0/87.2% (91.4/88.7%), respectively, for AC2RS-5 cm. This included oligomeric glucose/xylose concentrations of 0.54/1.61 g/L (0.07/1.50 g/L) and the respective glucan/xylan conversions of 4.8/24.6% (0.92/22.2%).

#### Kinetic modelling of AFEX pretreated rice straw

A dominant factor affecting the enzymatic hydrolysis rate is the severity of the pretreatment condition [42]. The increased severity of the AFEX C2 condition (log *R*_*o*_ = 2.88) made the pretreated rice straw substrate, particularly the larger particles, more susceptible to enzymatic hydrolysis than the AFEX C1 condition (log *R*_*o*_ = 1.48) and therefore increased the hydrolysis rate. The enzymatic hydrolysis data for UTRS, AC1RS and AC2RS substrates were fitted into the Chrastil diffusion-limited kinetic model based on Eq. (1) to further understand the kinetics of this unusual hydrolysis result. Table 2 summarizes the estimated kinetic parameters for UTRS, AC1RS and AC2RS substrates at 1% and 3% (not for UTRS) glucan loading hydrolysis. The parameters for each hydrolysis case were determined from experimental data using non-linear regression analysis. In all regression cases, a good agreement with the experimental results was obtained as indicated by coefficient of determination, R^2^ > 0.97 (Table 2). Therefore, the diffusion characteristics of the substrate-enzyme system in each hydrolysis case could be determined from the parameters *n* and *k*[44].

**Table 2:** Estimated parameters for substrate-enzyme diffusion-limited kinetic model for UTRS and AFEX pretreated rice straw at different particle size and glucan loading

**Substrate size**	**1% Glucan loading**					**3% Glucan loading**				
	**Diffusion-limited kinetic**			**Equilibrium sugar concentrations, *P***_***e ***_**at *t*** **= 168 h**		**Diffusion-limited kinetic**			**Equilibrium sugar concentrations, *P***_***e ***_**at *t*** **= 168 h**	
	***k *(L/g.h)**	***n***	**R**^**2 **^**(%)**	**Glucose (g/L)**	**Xylose (g/L)**	***k *(L/g.h)**	***n***	**R**^**2 **^**(%)**	**Glucose (g/L)**	**Xylose (g/L)**
UTRS – 2 mm	0.0475	0.293	98.5	2.9	0.6	-	-	-	-	-
UTRS – 5 cm	0.0309	0.285	99.0	2.7	0.5	-	-	-	-	-
AC1RS – 2 mm	0.0998	0.296	99.3	8.1	3.7	0.0300	0.364	99.2	24.4	9.9
AC1RS – 5 mm	0.1003	0.320	99.4	7.5	3.1	0.0286	0.340	99.5	21.8	8.7
AC1RS – 2 cm	0.1078	0.440	99.4	7.6	2.8	0.0288	0.444	99.2	22.0	8.2
AC1RS – 5 cm	0.0929	0.456	97.3	6.7	2.3	0.0282	0.509	99.5	19.9	7.0
AC2RS – 2 mm	0.0958	0.294	99.9	8.6	4.1	0.0184	0.397	98.9	22.1	10.1
AC2RS – 5 mm	0.1216	0.322	99.1	7.9	3.5	0.0182	0.381	98.0	20.3	8.6
AC2RS – 2 cm	0.1412	0.438	98.6	9.1	4.0	0.0323	0.528	99.5	27.0	10.6
AC2RS – 5 cm	0.1598	0.522	98.7	10.0	4.3	0.0345	0.616	98.4	29.4	11.4

The changes in the values of the structural diffusion resistance coefficient, *n*, show the progress of the modification of the substrates [23]. The *n* value for UTRS at 2 mm was higher than UTRS at 5 cm, indicating a smaller diffusion resistance for the former particle size, although the difference was not significant. Nonetheless, it is possible to use the *n* value and evaluate the extent of the structural modifications on the pretreated rice straw substrates for different AFEX pretreatment conditions and at different particle sizes. Obviously, increasing the particle size from 2 mm to 5 cm in the enzymatic hydrolysis at 1% glucan loading increased the *n* value for AFEX pretreated rice straw. The *n* value for AC1RS and AC2RS substrates ranged from 0.296 to 0.456 and from 0.294 to 0.522, respectively. Compared to UTRS, the change in *n* for AC1RS and AC2RS substrates yielded different scenarios for 2 mm and 5 mm particle sizes. While the *n* for the 2 mm substrate slightly changed from 0.293 in UTRS to 0.296 in AC1RS and 0.294 in AC2RS, the *n* for the 5 cm substrate increased from 0.285 in UTRS to 0.456 in AC1RS and 0.522 in AC2RS (Table 2). This change of *n* value suggests that the relevant changes in the diffusion and the structure of the substrate–enzyme system have occurred after pretreatment, resulting in less diffusion resistance in the pretreated samples [23]. Comparing the hydrolysis of AC1RS and AC2RS substrates, the smaller particle size (2 mm and 5 mm) for both substrate types produced almost similar *n* values ranging from 0.294 to 0.322 for 1% glucan loading hydrolysis, and from 0.340 to 0.397 for 3% glucan loading hydrolysis. A different *n* value was observed for larger particle sizes of AC1RS and AC2RS substrates (2 cm and 5 cm). The larger particle sizes of the AC2RS substrate expressed higher *n* values, 0.438 to 0.522, and 0.528 to 0.616 for 1% and 3% glucan loading hydrolysis, respectively, compared to AC1RS substrates (Table 2). This implied that severe AFEX pretreatment (AFEX C2 condition) using a larger particle size improved the diffusion of molecules in the pores of the substrate.

#### SEM histological changes of UTRS and AFEX pretreated rice straw epidermal surface

It has been reported that high digestibility of pretreated biomass is probably due to an increase in cellulose accessibility as a result of hemicellulose extraction and lignin redistribution [45,46]. In addition to the quantitative analysis of the hydrolysis trends of AC1RS and AC2RS, the SEM analysis provided further understanding of the hydrolysis of the AC1RS and AC2RS substrates based on histological changes of the UTRS and AFEX pretreated rice straw epidermal surface.

SEM images of UTRS at small particle size (2 mm and 5 mm) show that most of the cuticle and silica layers on the surface were already broken during the milling process and this greatly aided the AFEX C1 condition as the surface resistance was less than un-milled straw. Although it was a mild pretreatment condition, most of the papillae, cuticle and silica layers, and possibly lignin and other extractives, were easily cooked, melted and solidified in situ by the AFEX C1 condition, thereby exposing the cellulose fibers, making them more accessible to enzymes and ready for the subsequent hydrolysis. These degraded and solidified materials on the epidermal surface yielded a messy and compact surface, as indicated by the low *n* value in the kinetic model.

When the small particle size substrate (2 mm and 5 mm) was severely pretreated with the AFEX C2 condition, the severity of this pretreatment not only cooked and melted the papillae, cuticle and silica layers, but it also degraded the exposed cellulose fibers, producing poor hydrolysis performance (image not shown). Although the surface of AC2RS-5 mm was quite clean and less compact compared to the surface of AC1RS-5 mm, indicating the impact of high severity in the AFEX C2 condition, the hydrolysis of this substrate, at low or high glucan loading, normally yielded the lowest concentration and conversion due to cellulose degradation during pretreatment.

**Figure 5:**
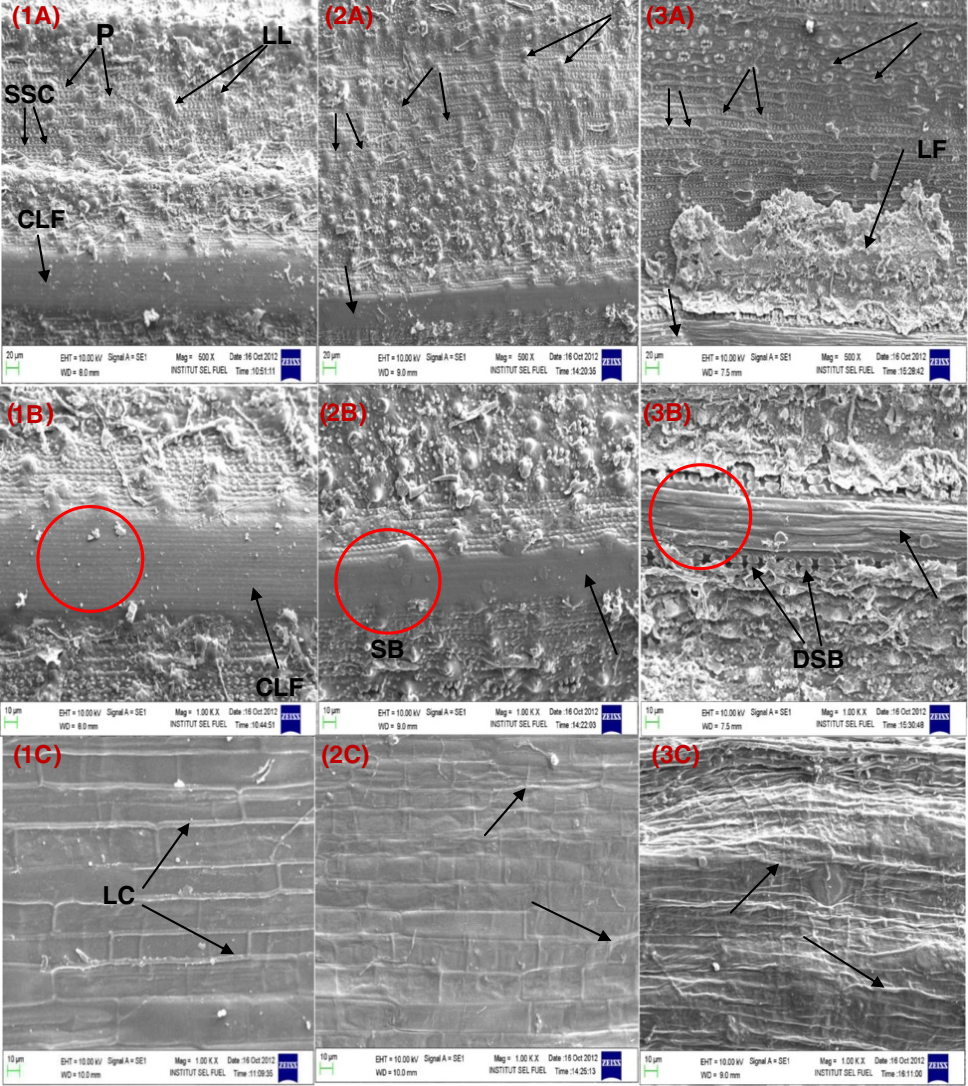
**SEM images of untreated rice straw (UTRS) and AFEX pretreated rice straw for 5 cm particles: 1A, B, C – Untreated rice straw; 2A, B, C – AFEX C1 pretreated rice straw (AC1RS); 3A, B, C - AFEX C2 pretreated rice straw (AC2RS).** Magnification of images given in Figure 1A, 2A and 3A are 500 X; while, all other images are magnification at 1000X. Details of the abbreviations given in the figure are: CLF – Cellulose large fibrils, DSB – Dumbbell silica body, LC – Long cells, LF – Large flake, LL – Large lump, P – Papillae, SB – Silica body, SSC – Silicified short cells.

Figure 5 shows the SEM images of the exterior epidermal surface of large particle size (2 cm and 5 cm) untreated rice straw (UTRS – 1A, B and C) and after AFEX pretreatment rice straw (AC1RS – 2A, B and C; AC2RS – 3A, B and C) samples. In AC1RS, some silica bodies were exposed on cellulose large fibrils due to removal of the cuticle layer by mild AFEX pretreatment (Figure 5(2B)), as silica is deposited as a layer beneath the cuticle layer [47]. While the cellulose configuration was still intact, some of the papillae structures were broken, showing the collapse of some cuticle layers, and the size of large lumps was also reduced. Most of the silicified short cells were still intact. Although the AFEX C1 condition could remove some cuticle layers, it was not adequate to make the cellulose more accessible to the enzymes. Poor hydrolysis was observed on AC1RS substrate with large particle size (2 cm and 5 cm).

SEM images show that AC2RS had a very clean and clear epidermal surface (Figure 5(3A)). Most of the papillae, cuticle and silica layers were diminished, and the large lumps together with lignin were deformed. These substances were condensed and agglomerated into large flakes (LF) which were redistributed on the particle surface resulting in a very clear view of the lump pits and twisted short cells. The cellulose fibers (CF) were clearly exposed to the surface with the dumbbell silica body (DSB) next to it indicating complete destruction and removal of the cuticle and silica layers. The absence of cuticle and silica layers, along with clean cellulose fibers, increased the cellulose accessibility to the enzymes, resulting in good digestibility and hydrolysis performance. This was also indicated by the high *n* and *k* values in the kinetic model of large particle of AC2RS substrates (2 cm and 5 cm). The interior epidermis of AC2RS also showed that the long cells were totally enlarged and started to disintegrate from the surface compared to UTRS and AC1RS substrates, resulting in higher digestibility (Figure 5(1C, 2C and 3C)). High severity pretreatment conditions (AFEX C2) in which the pretreatment temperature is 140°C, well above the glass transition temperature of lignin (120°C), (unpublished results) should have helped ammonia to solubilize lignin and re-deposit it on the surface when ammonia is removed after pretreatment.

#### Comparison of different glucan loading hydrolysis (1%, 3% and 6%)

**Figure 6:**
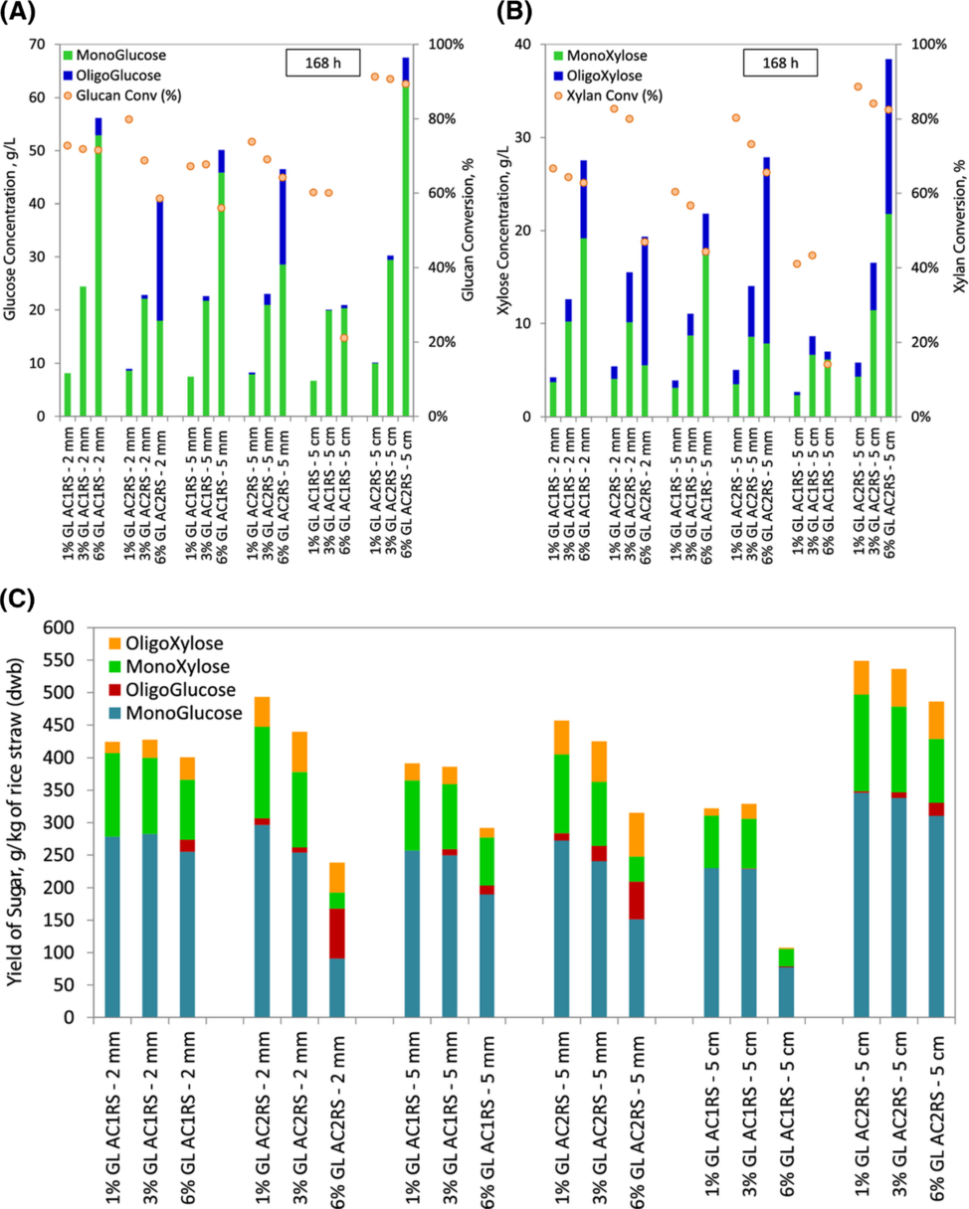
**Comparison on concentration, conversion and yield of sugar at different glucan loading for selected AFEX C1 (AC1RS) and AFEX C2 (AC2RS) biomass sizes.** (**A**) & (**B**) – Concentration and conversion, (**C**) – Yield.

Figure 6(A)/6(B) compares the glucan and xylan conversions after 168 h of hydrolysis from low to high glucan loading (1%, 3% and 6%) for AC1RS and AC2RS substrates at 2 mm, 5 mm and 5 cm. Theoretically, when the solid loading in the hydrolysis is increased, sugar concentrations should increase [48]. From the figure, it is clear that the concentrations of monomeric and oligomeric glucose/xylose for most substrates increased while glucan and xylan conversions decreased as the glucan loading increased from 1% to 6%. AC2RS-5 cm substrate continued to give the highest sugar concentrations (including the oligomeric sugars) and conversions at higher glucan loading. After 168 h hydrolysis, the glucose/xylose concentrations and glucan/xylan conversions were found to be 30.24/16.52 g/L and 90.7/84.1%, respectively, at 3% glucan loading and 67.47/38.38 g/L and 89.4/82.5%, respectively, at 6% glucan loading.

The combined effect of the pretreatment severity and mass transfer limitation were potentially affecting the performance of milled AC1RS and AC2RS substrates in the hydrolysis at higher glucan loading (3% and 6%). At 3% glucan loading hydrolysis, milled AC1RS and AC2RS substrates in their granular forms agglomerate easily when water is added, resulting in thick slurries of hydrolysis mixture which are difficult to uniformly mix [46]. The AFEX C1 condition (low severity) provided milder pretreatment to AC1RS substrates than the AFEX C2 condition, resulting in less LCC cleavage, less hemicellulose release and less lignin redistribution. Without good mixing this milder condition reduced the hydrodynamic interactions between particles and surrounding fluid as well as interaction among the particles and interfered less with enzyme diffusion [42,45,46]. Complemented with cellulose fibers that were successfully cleaned as well as perfectly exposed in milled AC1RS substrates which provided better enzyme accessibility, the hydrolysis of these substrates ultimately produced better monomeric sugar production than milled AC2RS. (Figure 6(A)/6(B)).

Even though at the macroscopic level milled AC2RS appeared to give similar slurry properties as milled AC1RS, the effect of AFEX C2 pretreatment severity also contributed to the complex slurry condition at the microscopic level. As discussed, the severity of this pretreatment degraded the cellulose fibers of AC2RS-5 mm, possibly together with LCCs and hemicelluloses, and thereby reduced the potential sugar availability in the substrate. In addition, there was more degradation and cleavage of chemical bonds, as well as lignin redistribution, in milled AC2RS substrate. Similar morphological changes and lignin globules were seen in corn stover as the AFEX severity was increased in corn stover [49]. This occurrence explained the low monomeric glucose and xylose concentrations at the end of 168 h hydrolysis of both milled AC2RS substrates (Figure 6(A)/6(B)).

Similar *n* values for smaller particle size of AC1RS and AC2RS substrates (2 mm to 5 mm) indicated that the diffusion resistance was approximately the same in both substrates. The *k* values in AC1RS-2 mm (0.0300 L/g.h) and AC1RS-5 mm (0.0286 L/g.h) substrates were much higher compared to AC2RS of the same size (0.0184 L/g.h for AC2RS-2 mm and 0.0182 L/g.h for AC2RS-5 mm) possibly due to cellulose fiber degradation in the latter substrates resulting in less cellulose hydrolysis (Table 2).

As observed during hydrolysis at 1% glucan loading, hydrolysis of AC1RS and AC2RS substrates at high glucan loading (3% and 6%) generally released higher concentrations of oligomeric xylose than oligomeric glucose, as shown in Figure 6(A)/6(B). The combination of Spezyme CP and Novozyme 188, used in the hydrolysis, could not efficiently hydrolyse the oligomeric xylose to monomeric xylose due to insufficient β-xylosidase activity in the enzyme preparations. Coupled with fast hydrolysis of xylan to oligomeric xylose, this led to the high concentration of oligomeric xylose and low concentrations of monomeric xylose, particularly in milled AC2RS substrates. This condition probably inhibited the cellulase activity and reduced the cellulose hydrolysis which led to high concentrations of oligomeric glucose in milled AC2RS substrates [43].

Hydrolysis of larger particle sizes of AC1RS and AC2RS substrates at 3% glucan loading showed a different trend compared to smaller particle sizes of (milled) AC1RS and AC2RS substrates at the same glucan loading. Both AC2RS-2 cm and AC2RS-5 cm substrates produced the highest glucose/xylose concentrations among all substrates at 3% glucan loading hydrolysis (Table 2). At low (1%) and high (3% and 6%) glucan loading hydrolysis, AC2RS-5 cm substrate behaved very differently than AC1RS-5 cm substrate. The former gave the highest glucose and xylose concentrations after 72/168 h of hydrolysis even at high solid loading (3% and 6% glucan loading) while the latter yielded the lowest sugar concentrations at all glucan loadings (Figure 6(A)/(B)). The substrate of AC2RS-5 cm completely disintegrated and solubilized into water and left only fine and “powdery-looking” particles, even when the solid loading of the hydrolysis was increased as previously shown (Figure 3). The Chrastil kinetic model indicates that for hydrolysis at 3% glucan loading, AC2RS-5 cm had the highest *n* and *k* values (0.616 and 0.0345 L/g.h) followed by AC2RS-2 cm substrates (0.528 and 0.0323 L/g.h) while AC1RS-5 cm gave low *k* value (0.0282 L/g.h) at a reasonably high *n* value (0.509). These kinetic parameters showed that the large particle size substrates, when severely pretreated with the AFEX C2 condition, actually had less diffusion resistance with increased catalytic hydrolysis properties, compared to the smaller particle size. This interpretation of hydrolysis kinetics was visually confirmed with SEM imaging analysis of AC2RS.

The combination of the AFEX C2 condition and large particle size of rice straw substrate indicated a different rheology and mass transfer system as compared to milled (small particle size) AC1RS and AC2RS substrates. When compared to milled rice straw, large particle size of AC2RS did not agglomerate under wet conditions and did not form thick slurries when water was added even at high solid loadings. Due to this phenomenon, free water was still available to facilitate the diffusion of cellulase and hemicellulase to the substrate in order for hydrolysis to occur. As the hydrolysis continued water retaining polymers, such as hemicellulose, are broken down resulting in increased levels of free water [42], producing a free flowing hydrolysate.

Figure 6(C) shows the total sugar yield for hydrolysis of AC1RS and AC2RS (2 mm, 5 mm and 5 cm) from low to high glucan loadings per dry weight of UTRS. The yield of monomeric glucose and xylose decreased while the yield of oligomeric glucose and xylose increased when the glucan loading increased from 1% to 6%. Among the substrates, AC2RS-5 cm demonstrated a consistent decreasing sugar yield as the glucan loading increased. Hydrolysis at 6% glucan loading revealed that the highest sugar yield was given by AC2RS-5 cm with a yield of 486.12 g/kg of rice straw equivalent to 76.0% of total theoretical maximum sugar yield with an average conversion of 85.9% from total glucan and xylan. On the other hand, AC1RS-5 cm gave the lowest sugar yield with only 107.6 g/kg of rice straw, about 16.8% of total theoretical maximum sugar yield, and equivalent to one-quarter of the AC2RS-5 cm sugar yield. As for AC1RS substrates, hydrolysis at 6% glucan loading indicated that AC1RS-2 mm also could produce reasonable sugar yields with 400.6 g/kg of rice straw.

## Conclusions

Two AFEX pretreatment conditions of different severities were used to pretreat different particle sizes of rice straw, from milled substrates (2 mm and 5 mm) to cut substrates (2 cm and 5 cm). For either milled or cut rice straw, AC2RS substrates always gave higher sugar concentrations and conversions when compared to AC1RS substrates of the same size, demonstrating the greater effectiveness of AFEX C2 condition. While AC1RS substrates showed declining sugar conversion trends as the size of milled and cut substrates increased, AC2RS substrates demonstrated opposite sugar conversion trends between milled and cut substrates. As with milled AC1RS substrates, milled AC2RS substrates also showed a decreasing sugar conversion trend as the particle size increased. Cut AC2RS substrates exhibited an increasing sugar conversion trend when the substrate size increased, which has never been reported in the literature, at least to our knowledge. While the AC1RS-5 cm substrate hydrolysed slowly and solids remained intact with minor physical disintegration, the AC2RS-5 cm substrate completely disintegrated after the same period of hydrolysis and only left fine particles in the hydrolysate. The Chrastil diffusion-limited kinetic model was able to model the experimental data and explain the hydrolysis behaviour at different particle size based on kinetic parameters, *k* and *n*. Analysis of SEM imaging supported our interpretation of the experimental hydrolysis behaviour and kinetic data.

## Methods

**Figure 7:**
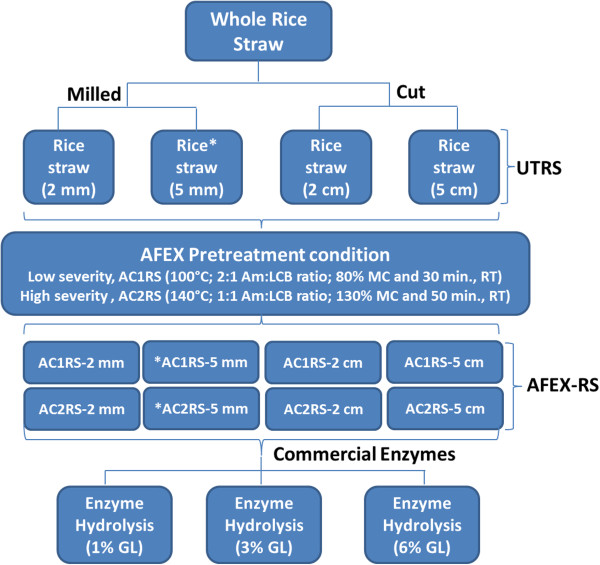
**Process flow diagram showing how biomass was processed, pretreated and hydrolysed at different glucan loadings.** Biomass that was subject to composition analysis is shown as (*). Here, Am - ammonia; LCB - lignocellulosic biomass; MC - moisture content; RT – residence time; GL – glucan loading; UTRS – untreated rice straw; AFEX-RS – Ammonia fiber expansion pretreated rice straw.

A process flow diagram showing how rice straw was processed to different particle sizes, pretreated by two AFEX pretreatment conditions of different severities and hydrolysed at three different glucan loadings is given in Figure 7.

### Feedstock

Rice straw from a medium-grain rice crop obtained from the central part of Selangor, Malaysia was used as the feedstock. It was air-dried to 10% moisture content (dry weight basis of biomass, dwb). Some of the rice straw was milled using a Foss mill (Eden Prairie, MN) and passed through 2 mm and 5 mm screens, while other samples were manually processed using scissors to 2 cm and 5 cm long. All processed rice straw samples were labelled as 2 mm, 5 mm, 2 cm and 5 cm and were stored at 4 °C until further use.

### AFEX pretreatment

Two statistically optimized AFEX pretreatment conditions from a previous study, identified as AFEX C1 and AFEX C2, were used to pretreat the rice straw [50]. Table 3 presents the details of the AFEX pretreatment conditions used. The logarithm of the reaction ordinate (log *R*_*o*_) is defined as the severity of the pretreatment, where the reaction ordinate is given:

(2) $$R_{o}=t\;x\;e^{\left[\frac{\left(T_{r}-T_{b}\right)}{14.75}\right]}$$

where *t* is the residence time (min), *T*_*r*_ is the pretreatment temperature (°C), *T*_*b*_ is the base temperature (100°C) and 14.75 is the activation energy. Biomass of the predetermined moisture level was loaded into a bench-top high-pressure Parr reactor with a 2000 mL capacity (PARR Instrument Co., IL) and liquid ammonia was slowly charged to the reactor. The reactor temperature was raised and maintained at the desired temperature for a given residence time and pressure, as reported before [1]. AFEX C1 pretreated rice straw materials of different particle sizes were labeled as: AC1RS-2 mm, AC1RS-5 mm, AC1RS-2 cm, AC1RS-5 cm, while AFEX C2 pretreated rice straw were labeled as: AC2RS-2 mm, AC2RS-5 mm, AC2RS-2 cm and AC2RS-5 cm. All pretreated samples were dried under a fume hood overnight to remove residual ammonia and were then placed in zip-locked bags and stored at −20°C until further use.

**Table 3:** Conditions for AFEX C1 and AFEX C2 used in rice straw pretreatment

**AFEX parameters**	**AFEX C1**	**AFEX C2**
Pretreatment temperature, *T*_*r*_ (°C)	100	140
Ratio of ammonia to rice straw (w/w)	2:1	1:1
Ratio of moisture to rice straw (w/w, dwb)	80%	130%
Residence time, *t* (min)	30	50
Severity, log *R*_*o*_	1.48	2.88

### Compositional analysis

Compositional analysis was performed on untreated rice straw (UTRS) and AFEX pretreated rice straw (AC1RS and AC2RS using milled rice straw of 5 mm particle size) according to Laboratory Analysis Protocol (LAP) developed by the National Renewable Energy Laboratory (Golden, Colorado USA) [51-53]. The UTRS and AFEX pretreated rice straw (AC1RS and AC2RS) were extracted with water and 95% ethanol using an ASE2000 (Accelerated Solvent Extractor, DIONEX, CA) to remove the extractives before quantifying the structural carbohydrates and lignin in the acid hydrolysis step. Crude protein was calculated based on nitrogen content in the biomass. A Skalar Primacs SN Total Nitrogen Analyser (Breda, Netherlands), was used to estimate the nitrogen content in the biomass using the Dumas method.

### Enzymatic hydrolysis

Enzymatic hydrolysis of UTRS and AFEX pretreated rice straw was performed according to the Laboratory Analysis Protocol (LAP 009) developed by the National Renewable Energy Laboratory [54]. The hydrolysis was carried out at low (1%) and high (3%) glucan loading (w/v) in a 15 mL reaction volume using 20 mL scintillation vials and 50 mL Falcon tubes, respectively. Higher glucan loading (6% (w/v), equivalent to 17% of solid loading on dry weight basis) was conducted in a 300 mL reaction volume using a 2000 mL Erlenmeyer flask.

The enzyme mixture consisted of Spezyme® CP (Batch no: 4900901224) from Genencor International (Rochester, NY) and Novozyme™ 188 (Batch no: 078 K0709) from Sigma-Aldrich Co. (St. Louis, MO). The hydrolysis samples of 1%, 3% and 6% glucan loading were mixed with the desired cellulase enzymes at 15 FPU/g glucan (protein concentration 123 mg/ml) and a *β*-glucosidase enzyme loading of 64 *p*NPGU/g glucan (protein concentration of 168 mg/ml). The hydrolysis reaction for 1% and 3% glucan loading was carried out at 50°C, 150 rpm, and pH 4.8 using 1 M citrate buffer. Tetracycline (40 mg/L) and cyclohexamide (30 mg/L) were added as antibiotic agents in the hydrolysis samples. For 6% glucan loading, the hydrolysis reaction was carried out at the same temperature and pH with a shaker speed of 250 rpm to achieve good mixing performance. Chloramphenicol (50 mg/L) was added to the 6% glucan loading sample as antimicrobial agent to minimize the risk of contamination [55]. Hydrolysate samples for the 1% and 3% glucan loading experiments were taken at specified time intervals (4 h, 8 h, 12 h, 24 h, 48 h, 72 h and 168 h), placed in capped micro-centrifuge tubes, heat-treated at 100°C for 20 minutes on a heating block (to denature the enzyme), centrifuged at 4400 rpm for 10 minutes and then filtered through a 0.22-µm Whatman membrane syringe filter. The 168 h 6% glucan loading hydrolysate was centrifuged twice at 6000 rpm and then 10000 rpm to separate the hydrolysate from the un-hydrolysed solids [55].

### HPLC analysis for monomeric sugars

All All monomeric sugars (glucose, xylose and arabinose) were analyzed using high performance liquid chromatography (HPLC). The HPLC system consists of a Shimadzu LC-2010 (Milford, MA) equipped with a Waters 410 refractive index detector. An Aminex HPX-87P column (Bio-Rad, Sunnyvale, CA, USA) with a de-ashing guard cartridge (Bio-Rad) was used for monomeric sugars concentration analysis in hydrolysate. Degassed HPLC grade water was used as the mobile phase at 0.6 ml/min at a column temperature of 85°C. An Aminex HPX-87H column (Bio-Rad, Sunnyvale, CA, USA) was used to quantify the sugar concentrations in the acid hydrolysis samples for compositional and oligomers analysis. 5 mM sulfuric acid (H_2_SO_4_) was used as the mobile phase at 0.6 ml/min at a column temperature of 50°C. The HPLC sample injection volume was 10 µl. Standard curves were generated using different concentrations of mixed sugars [1].

### Sugar conversion and yield

AFEX pretreatment is a dry to dry process, and therefore the sugar recovery after AFEX C1 and AFEX C2 depended primarily on the pretreated solid recovered after the pretreatment. The sugar conversion after enzymatic hydrolysis was calculated using the actual sugar produced in the hydrolysis over the available theoretical sugar in the rice straw, while the sugar yield was calculated using the actual mass of total sugar produced over the actual mass of UTRS (dwb) used in the hydrolysis. Below are the equations used in the calculations:

(3) $$\begin{array}{c} \text{Overall}\;\text{sugar}\;\text{conversion}\;\left(\%\right)\\ =\frac{\left(C_{\text{Mono}}+C_{\text{Oligo}}\right)xV}{\text{TSC}} \end{array}$$

(4) $$\text{Overall}\;\text{sugar}\;\text{yield}\;\left(\%\right)=\frac{\left(C_{\text{Mono}}+C_{\text{Oligo}}\right)xV}{W_{\text{URS}}}$$

where *C*_*Mono*_ and *C*_*Oligo*_ are the monomeric and oligomeric sugar concentrations in g/L, *V* is the volume of enzymatic hydrolysis reaction in L, *TSC* is the theoretical sugar content in the hydrolysis at specified glucan loading in g, and *W*_*UTRS*_ is the weight of the UTRS in kg (dwb).

### Kinetic modeling and parameter estimation

The experimental data on enzymatic hydrolysis of UTRS (5 mm and 5 cm), AC1RS (2 mm, 5 mm, 2 cm and 5 cm) and AC2RS (2 mm, 5 mm, 2 cm and 5 cm) substrates at 1% and 3% glucan loading were fitted according to Eq. (1). The parameters *k* and *n* of the model were determined using the Generalized Reduced Gradient (GRG) Nonlinear algorithm in Microsoft Excel Solver.

### Scanning Electron Microscopy (SEM) imaging of UTRS and AFEX pretreated rice straw

Scanning electron microscopy (SEM: ZEISS-EVO MA 10, UK, EDX: EDAX-APOLLO X, USA) studies were conducted to examine the histological changes on the exterior and interior epidermis of rice straw after AFEX pretreatment. All samples were coated with a thin layer of gold using sputter coater (QUORUM Q150RS, UK). The samples were then mounted carefully on the SEM stub and gently pressed.

## Abbreviations

AFEX: Ammonia Fiber Expansion; AC1RS: AFEX C1 pretreated rice straw; AC2RS: AFEX C2 pretreated rice straw; AGX: Arabino-glucuronoxylan; FA: Ferulic Acid; FPU: Filter paper unit; GRG: Generalized Reduced Gradient; LAP: Laboratory Analysis Protocol; LCB: Lignocellulosic biomass; LCC: Lignin carbohydrate complex, MC, Moisture content; MeGlcA: 4-O-methyl-α-D-glucopyranosyl uronic acid; pNPGU: p-nitrophenyl-β-D-galactopyranoside; TSC: Theoretical sugar content; UTRS: Untreated rice straw.

## Competing interests

The authors declare that they have no competing interests.

## Authors’ contribution

SH and VB designed the pretreatment and hydrolysis experimental conditions and drafted the manuscript. SH performed the pretreatment, hydrolysis and performed statistical analysis. BD participated in the design and coordination of the study. All authors provided critical input to the manuscript and read and approved the final manuscript.
